# Parent-Reported Orofacial Health in Preterm- and Term-Born Children: An Exploratory Cross-Sectional Study

**DOI:** 10.3390/children13101349

**Published:** 2026-10-05

**Authors:** Małgorzata Łysiak-Majchrzak, Izabela Czołgosz, Teresa Matthews-Brzozowska

**Affiliations:** Department of Maxillofacial Orthopaedics and Orthodontics, Ludwik Rydygier Collegium Medicum in Bydgoszcz, Nicolaus Copernicus University in Toruń, 87-100 Toruń, Poland

**Keywords:** preterm birth, feeding, breastfeeding, bottle feeding, pacifier, orofacial function, malocclusion, children

## Abstract

**Highlights:**

**What are the main findings?**

- Feeding duration and birth status showed no FDR-robust associations.
- Longer pacifier use was linked to parent-reported perioral/smile abnormality.

**What are the implication of the main finding?**

- The observed association between pacifier use duration and parent-reported orofacial outcomes should be examined in future studies using standardized clinical assessments.
- Clinical assessment is needed to confirm these parent-reported findings.

**Abstract:**

Background/Objectives: Early feeding experiences and non-nutritive sucking habits coexist with the development of orofacial functions and occlusion. This study compared the duration of breastfeeding, bottle feeding, and mixed feeding, as well as parent-reported orofacial outcomes in preterm- and term-born children, and exploratorily assessed associations among these variables. Methods: A cross-sectional parent/caregiver survey used open convenience recruitment in online prematurity-related groups. The main comparison included 108 children (88 preterm and 20 term). Unambiguous duration records for all three feeding modes were available for 92 children and were analyzed as separate quantitative variables. Results: Among these 92 children, median breastfeeding duration was 6 months (IQR 2–12), bottle-feeding duration was 0 months (IQR 0–12), and mixed-feeding duration was 0 months (IQR 0–5). No preterm–term differences were found for breastfeeding (*p* = 0.090), bottle feeding (*p* = 0.357), or mixed feeding (*p* = 0.586). Each additional 12 months of pacifier use was associated with higher odds of a parent-reported perioral/smile abnormality (aOR = 2.29; 95% CI 1.25–4.19; *p* = 0.007; q = 0.028). Conclusions: Longer reported pacifier use was associated with a broad parental perception of smile abnormality after FDR correction; causality and clinical significance cannot be established. The findings require confirmation using clinically verified perinatal data and standardized orofacial assessment.

## 1. Introduction

Development of the stomatognathic system reflects repeated functional activity and the interaction of neuromotor factors during anatomical maturation. Sucking, swallowing, breathing, chewing, and later speech articulation engage the muscles of the lips, tongue, cheeks, and mandible; disturbances in these functions may coexist with myofunctional and occlusal abnormalities [1,2,3]. Children with perinatal vulnerabilities, particularly those born preterm or with very low birth weight, may also exhibit distinct orofacial characteristics and altered craniofacial maturation [4,5,6].

Feeding is one of the earliest functional stimuli. Breastfeeding, bottle feeding, and mixed feeding involve different sucking patterns and muscle activity, and systematic reviews indicate possible associations between feeding mode or duration and selected occlusal traits [7,8]. In preterm-born children, feeding history may additionally be shaped by immature suck–swallow–breathe coordination, respiratory needs, and temporary non-oral feeding [1,4,9,10,11].

Pacifier use is common in early childhood. Available evidence suggests that duration and the pattern of non-nutritive sucking may be more relevant to potential occlusal consequences than use alone [12,13,14]. Other parafunctions may likewise coexist with myofunctional disorders and particular occlusal traits [2,13,15].

The literature on later stomatognathic consequences of prematurity is heterogeneous. Comparisons require careful attention to population definitions because many studies enrolled very preterm children or children with low or very low birth weight, who have greater perinatal vulnerability [4,16]. Studies covering a broader prematurity spectrum have not consistently confirmed preterm birth as an independent risk factor [9,17,18,19]. The contemporary concept of perinatal vulnerability emphasizes that gestational age, birth weight, the neonatal course, nutrition, and intensive-care exposures may all be relevant [5]. The environment in which a child develops may also contribute [20].

Population data show that preterm birth remains an important public-health issue in Poland. According to Statistics Poland, 16,815 live births in 2024 occurred before 37 completed weeks of gestation, representing 6.68% of all live births [21].

The studies discussed above address selected relationships between prematurity, feeding, non-nutritive sucking, and clinical occlusal outcomes [4,7,8,12,13,14,16,17,18,19]. They provide less information on how separately recorded breastfeeding, bottle-feeding, and mixed-feeding durations coexist with a range of parent-reported functional and oral-health outcomes in a single sample containing both preterm- and term-born children. The present study provides an exploratory description of these variables within the same survey framework. It enriches the clinical literature with caregiver-reported perspectives and highlights the need for longitudinal studies and standardized clinical assessments.

The primary objective was to compare two sets of variables between children reported as born preterm and those reported as born at term: (i) the durations of breastfeeding, bottle feeding, and mixed feeding, measured separately in months; and (ii) the frequencies of parent-reported orofacial outcomes, defined in Section 2.6. Secondary exploratory analyses examined associations of feeding duration and pacifier exposure with these outcomes. Feeding duration and pacifier use were exposure variables; functional difficulties, reported dental problems, perceived smile abnormality, and oral-health ratings were outcome variables. The cross-sectional design allows the identification of associations, while causal inference requires longitudinal evidence.

## 2. Materials and Methods

### 2.1. Study Design and Participants

This cross-sectional survey was completed by parents or caregivers, who reported information on the child’s perinatal period, feeding history, selected orofacial functions, parafunctions, and dental problems. Data were collected by computer-assisted web interviewing via Typeform platform (Typeform S.L., Barcelona, Spain) from 1 June to 31 July 2026. Recruitment was open, convenience-based, and supplemented by snowball sampling. The survey link was distributed through Facebook groups run by two major Polish foundations concerned with prematurity. This channel was intentionally effective for reaching parents of preterm-born children and therefore overrepresented this group. The term-born group was consequently a convenience comparison group rather than a population-matched control group. Because the invitation was openly distributed, the number of people who received or viewed the link could not be determined reliably; a conventional response rate was therefore not calculated. This sampling approach precludes estimation of population prevalence and direct comparison of the preterm/term proportions with registry data. Selection into these online groups and voluntary participation may depend on perinatal history, parental concerns, or health-related engagement. The resulting groups may differ in these characteristics as well as age. Consequently, between-group estimates describe this recruited sample and cannot be generalized to all preterm- and term-born children. Potential recruitment-related selection bias should be considered when interpreting the age- and sex-adjusted findings.

Reporting of the online survey procedures was checked against selected CHERRIES and CROSS items, particularly recruitment, informed consent, limitations on calculation of the response rate, and identification of potentially duplicate entries [22,23]. The survey was anonymous and required neither personal identifiers nor mandatory login. Eligibility required provision of informed consent for anonymous participation.

### 2.2. Questionnaire Development and Variables

No standardized oral-health-related quality-of-life (OHRQoL) instrument was used. The authors developed a questionnaire covering parent-reported perinatal characteristics, feeding history, orofacial functions, dysfunctions, parafunctions, perioral/smile abnormalities, and oral health. Development comprised a review of the relevant subject areas, qualitative expert assessment, and pilot testing.

The preliminary questionnaire was qualitatively reviewed by three purposively selected experts with complementary expertise: two in dentistry/orthodontics and one in survey methodology. Pilot testing included 40 respondents: parents of 20 preterm-born and 20 term-born children. Pilot data were not included in the main analysis. Expert review was qualitative; no formal content validity index was calculated. Pilot testing evaluated question clarity, the technical survey pathway, and response completeness rather than the instrument’s psychometric validity.

The exact item on the principal endpoint asked, “Czy zauważasz nieprawidłowości w uśmiechu swojego dziecka?” (“Do you notice any abnormalities in your child’s smile?”). The question did not specify crowding, open bite, asymmetry, or other examples, and no standardized grading or clinical examination was performed. We therefore refer to this response as a parent-reported perioral/smile abnormality and do not infer a specific phenotype.

### 2.3. Analytical Eligibility and Data Cleaning

**Figure 1:**
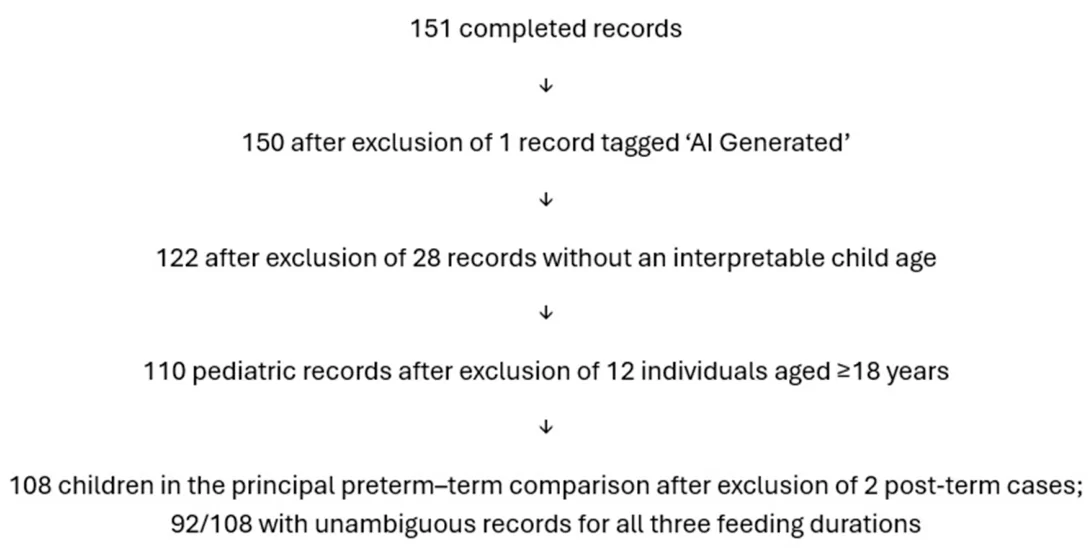
Participant record selection and analytical samples.

The dataset contained 151 completed records. One record tagged as ‘AI Generated’ was excluded, followed by 28 records without an interpretable age and 12 records concerning individuals aged ≥18 years. This left 110 pediatric records: 88 reported as preterm, 20 as term, and 2 as post-term. The two post-term cases were excluded from the principal preterm–term comparison, leaving 108 children (Figure 1).

No a priori sample-size or power calculation was performed. The final sample was determined by eligible responses during the recruitment period; analyses are exploratory and may have limited power, especially for comparisons involving 20 term-born children and rare outcomes.

### 2.4. Birth-Status Classification and Birth Weight

Birth status was determined solely from the parent’s categorical response indicating whether the child had been born preterm, at term, or post-term. Preterm birth was defined as birth before 37 completed weeks of gestation. Reliable, complete exact gestational-age information was not available for validation, and birth records or other medical documentation were not obtained. Agreement between the reported category and a clinical reference therefore could not be assessed. Misclassification may have obscured or distorted between-group differences, and its direction and magnitude cannot be quantified from these data. The findings apply to broadly defined parental birth-status categories; reliable subdivision into moderate/late, very, or extremely preterm groups was not possible.

Birth weight was considered an additional exploratory perinatal characteristic.

### 2.5. Feeding and Oral-Habit Exposures

The exposures were tube feeding after birth; breastfeeding duration; bottle-feeding duration; mixed-feeding duration; and pacifier use. The categorical feeding-mode response was retained for descriptive purposes, whereas the main feeding analysis treated the duration of each mode as a separate quantitative variable in months. The duration field was structured as breast–bottle–mixed. Quantitative analysis included only records in which all three values could be read unambiguously; 92/108 children (85.2%) met this condition. A value of 0 indicated no exposure to that feeding mode. Because the distributions were strongly skewed, the median and IQR were the primary descriptive measures. Tube feeding was very rare and was treated descriptively.

Incomplete or ambiguous values were not imputed. Each comparison used the participants with usable data for the variables in that analysis, so denominators vary across outcomes. Requiring unambiguous durations for all three feeding modes excluded 16/108 children from the quantitative feeding analyses; if missingness is related to exposure or outcome, selection bias is possible.

### 2.6. Orofacial Outcomes

The parent-reported outcomes comprised feeding, swallowing, or sucking difficulties; articulation difficulties; occlusion or tooth-position problems; a reported diagnosis of malocclusion; dysfunctions involving breathing, swallowing, chewing, or tongue position; parafunctions; perioral/smile abnormalities; and oral health rated as abnormal. These responses were not regarded as equivalent to a standardized clinical examination.

### 2.7. Statistical Analysis

Categorical variables are presented as counts and percentages, and continuous variables as means with standard deviations and/or medians with interquartile ranges, as appropriate. Age and exposure durations were compared between groups using the Mann–Whitney U test, and binary variables using Fisher’s exact test. Breastfeeding, bottle-feeding, and mixed-feeding duration constituted the principal quantitative description of feeding exposure. Separate models were constructed for each feeding mode and adjusted for the child’s current age, sex, and preterm/term status; adjusted odds ratios (aORs) were reported per additional 6 months of exposure.

Because some outcomes were rare, the groups were unequal, and complete or quasi-complete separation was possible, all adjusted binary models used Firth bias-reduced logistic regression (penalized likelihood) [24]. The Benjamini–Hochberg procedure was used to control the false discovery rate (FDR). Analyses were conducted in Statistica 13.3. Statistical significance was set at *p* < 0.05; q < 0.05 was considered robust to multiple testing.

Current age was included because outcome recognition and dental development vary with age and the birth-status groups differed in age; sex was included as a basic demographic covariate. Birth status was included in feeding-duration models to account for the sampling design and a potential relationship with exposure and outcome. Birth-status models were adjusted for age and sex; pacifier-duration models were adjusted for age and sex. Covariate adjustment does not remove confounding from unmeasured gestational severity, birth weight, socioeconomic circumstances, or clinical history. Unadjusted group comparisons in Table 1 and Table 2 remain descriptive and do not correct for the age imbalance.

## 3. Results

### 3.1. Characteristics of the Analytical Sample

The principal comparison included 108 children: 56 girls (51.9%) and 52 boys (48.1%). Eighty-eight children were classified as preterm and 20 as term. Preterm-born children were older at the time of the survey than term-born children (11.69 ± 3.39 vs. 8.80 ± 3.73 years; *p* = 0.002), whereas the sex distribution did not differ significantly (*p* = 0.136).

### 3.2. Early Feeding and Pacifier Use

The main analysis of feeding history was based on the duration of the individual feeding modes. An unambiguous record of breastfeeding, bottle-feeding, and mixed-feeding duration was available for 92/108 children (85.2%): 76 preterm and 16 term. In this group, median breastfeeding duration was 6 months (IQR 2–12), bottle-feeding duration was 0 months (IQR 0–12), and mixed-feeding duration was 0 months (IQR 0–5). Positive durations were recorded for breastfeeding in 74/92 children, bottle feeding in 32/92, and mixed feeding in 30/92. Term-born children had a numerically longer breastfeeding duration than preterm-born children (9 [6–17.25] vs. 6 [1–12] months), but the difference was not significant (*p* = 0.090). No differences were found for bottle-feeding duration (*p* = 0.357) or mixed-feeding duration (*p* = 0.586).

Tube feeding was reported for 1/87 preterm-born children (1.1%) and 1/20 term-born children (5.0%; *p* = 0.340). Pacifier use was reported for 60/87 preterm-born children (69.0%) and 11/20 term-born children (55.0%; *p* = 0.295). Among users, an unambiguous numerical duration was available for 54 preterm-born and 10 term-born children; the corresponding medians were 18 months (IQR 12–24) and 15 months (IQR 12–24), respectively (*p* = 0.505).

### 3.3. Feeding Duration as a Quantitative Exposure and Orofacial Outcomes

Separate Firth models estimated the change in outcome odds per additional 6 months of breastfeeding, bottle feeding, or mixed feeding, adjusted for current age, sex, and preterm/term status. None of the 23 models retained q < 0.05 after Benjamini–Hochberg correction. The strongest nominal signals involved mixed-feeding duration: articulation difficulties (aOR = 1.86; 95% CI 1.17–2.96; *p* = 0.008; q = 0.194), occlusion/tooth-position problems (aOR = 1.70; 95% CI 1.04–2.78; *p* = 0.033; q = 0.319), and diagnosed malocclusion (aOR = 1.65; 95% CI 1.02–2.66; *p* = 0.042; q = 0.319). These findings should be treated as exploratory signals, not confirmed associations.

### 3.4. Parent-Reported Orofacial Outcomes by Birth Status

In the primary comparison of parent-reported outcomes, age- and sex-adjusted Firth models showed that none of eight outcomes met the q < 0.05 threshold after Benjamini–Hochberg correction. Orofacial dysfunction was reported in 6/87 preterm-born and 6/19 term-born children (aOR = 0.15; 95% CI 0.04–0.61; *p* = 0.008; q = 0.061); occlusion/tooth-position problems were reported in 45/85 and 14/20, respectively (aOR = 0.23; 95% CI 0.07–0.80; *p* = 0.021; q = 0.083) (Table 3). These estimates are imprecise and their directions must not be read as evidence of protection from preterm birth, given the small convenience comparison group, age difference, and subjective outcomes.

### 3.5. Pacifier Duration and Orofacial Outcomes

After harmonizing pacifier duration and assigning 0 months to children with an unambiguous report of no pacifier use, the smile-outcome model included 97 children with complete data. Each additional 12 months of exposure was associated with higher odds of the parent-reported abnormality after adjustment for age and sex (aOR = 2.29; 95% CI 1.25–4.19; *p* = 0.007; q = 0.028). This endpoint was a broad parental perception, without clinical assessment or standardized grading; the specific perceived abnormality cannot be identified from the response. The estimate describes a cross-sectional association and does not establish a causal effect. Analogous models were not significant after FDR correction for diagnosed malocclusion (aOR = 1.27; *p* = 0.286; q = 0.381), occlusion/tooth-position problems (aOR = 1.29; *p* = 0.240; q = 0.381), or orofacial dysfunction (aOR = 1.03; *p* = 0.913; q = 0.913) (Table 4).

### 3.6. Exploratory Within-Cohort Associations

In secondary within-cohort analyses using adjusted Firth regression, parent-reported perioral/smile abnormality co-occurred with oral health rated as abnormal (aOR = 9.49; 95% CI 2.32–38.81; *p* = 0.002; q = 0.012). The estimate for ever having used a pacifier was extremely imprecise because of a zero cell (aOR = 17.98; 95% CI 1.08–300.46; *p* = 0.044; q = 0.102) and did not survive FDR correction; it should not be interpreted as a reliable effect estimate.

## 4. Discussion

### 4.1. Principal Findings

The comparison included 88 preterm-born and 20 term-born children, with the sample structure reflecting recruitment primarily through prematurity-foundation groups. Feeding history was described quantitatively. Positive mixed-feeding duration was recorded for 30/92 children, and the IQR in the analyzed group was 0–5 months. No preterm–term differences were identified in breastfeeding, bottle-feeding, or mixed-feeding duration. In adjusted Firth models, no association between feeding duration and orofacial outcomes was robust to FDR correction. Nominal signals involving longer mixed feeding and articulation or occlusion require independent confirmation and should not be interpreted as evidence of a causal effect of feeding mode.

After Firth regression and correction for multiple comparisons, none of the eight parent-reported orofacial outcomes showed a robust difference between birth-status groups. The nominal direction for orofacial dysfunction was unexpected, but rests on only six events in each group and a small, younger, nonrepresentative term-born comparison group. No protective effect of preterm birth can be inferred.

Within this sample, the association between reported pacifier-use duration and parent-reported perioral/smile abnormality met the FDR-adjusted threshold. The co-occurrence of this response with an abnormal parental oral-health rating also met that threshold. These are exploratory statistical associations involving broad, non-standardized parental measures; they do not establish reproducibility, clinical validity, or a specific orthodontic phenotype.

### 4.2. Preterm Birth and Early Feeding History

Differences in the course and duration of feeding among preterm-born children are biologically plausible in light of immature suck–swallow–breathe coordination and the more frequent need for neonatal feeding support [10]. Observational evidence also indicates that preterm-born and term-born children may differ in feeding patterns during the first years of life [11]. In our sample, median breastfeeding duration was shorter in preterm-born children than in term-born children (6 [1–12] vs. 9 [6–17.25] months), although this difference was not statistically significant. Nominal associations involving mixed feeding require caution because the analyses were exploratory and feeding modes were interdependent.

Interpretation also requires caution because birth status was survey-reported. Preterm/term status was based on a categorical parental response, without a separate reliable variable for the exact gestational week or independent verification from medical records.

### 4.3. Birth Status, Occlusion, and Orofacial Function

The association between prematurity and malocclusion remains inconsistent, and comparison across studies requires consideration of both the degree of prematurity and birth weight. Primožič et al. studied prematurely born children with low birth weight, Maaniitty et al. examined very preterm children, and Rocha Ruiz et al. focused on very-low-birth-weight preterm infants [4,16,25]. These populations had greater perinatal vulnerability than a broadly defined group of all preterm-born children. Da Rosa et al. reported a higher prevalence of moderate or severe primary-dentition malocclusion, with the association modified by breastfeeding and pacifier use [18]. Previous findings should not be interpreted as evidence of uniform orthodontic risk across the prematurity spectrum. Objois and Gebeile-Chauty found a higher prevalence of bilateral crossbite among preterm-born children, whereas increased impacted teeth and the greatest orthodontic treatment need were observed mainly in very and extremely preterm children [17]. The degree of prematurity may therefore be more important than a simple preterm–term dichotomy.

Similarly, in a cohort of 205 children born before 32 weeks and/or with birth weight <1500 g, Maaniitty et al. found nearly threefold higher odds of crowding in the primary dentition (OR = 2.94) and higher odds of previous orthodontic treatment in the mixed dentition (OR = 2.80) [16]. These findings further support considering gestational age and birth weight separately when comparing prematurity studies.

The nominal direction for reported orofacial dysfunction should be interpreted with particular caution: the term-born group contained only 20 children, was younger, and was recruited through prematurity-related channels. The age difference (11.69 versus 8.80 years on average) may also reflect different stages of mixed-to-permanent dentition and different opportunities for parents to notice tooth position or smile changes. Age adjustment in regression cannot fully account for differences in developmental stage or residual confounding.

### 4.4. Pacifier Use: Duration Rather than a Simple Yes/No Classification

Each additional 12 months of reported pacifier use was associated with higher odds of a parent-reported perioral/smile abnormality after adjustment for age and sex (aOR = 2.29; 95% CI 1.25–4.19; q = 0.028). The question did not define the abnormality or provide examples, so the response cannot distinguish perceived crowding, open bite, asymmetry, other dental or perioral features, or a general aesthetic concern. Its clinical relevance is therefore unresolved, and the estimate cannot be translated into an orthodontic diagnosis, severity rating, treatment need, or recommendation about pacifier cessation. Corresponding associations with reported diagnosed malocclusion, tooth-position problems, and orofacial dysfunction did not survive FDR correction. Retrospective reporting by the same caregiver permits recall and common-method bias, and residual confounding remains possible. This finding generates a hypothesis for prospective studies using clearly defined, clinically assessed outcomes; it does not demonstrate that pacifier use causes an abnormality.

### 4.5. Smile and Parent-Reported Oral Health

A secondary within-cohort association was observed between parent-reported perioral/smile abnormality and oral health rated as abnormal. Although malocclusion may affect oral-health-related quality of life [26], neither response in this survey establishes malocclusion or measures OHRQoL with a validated instrument. Their co-occurrence may partly reflect overlapping parental perceptions and a shared reporting source. The wide confidence interval supports cautious interpretation as an exploratory association.

### 4.6. Strengths and Limitations

This study has several strengths. It links perinatal and early-feeding information with a broad range of parent-reported functional and orthodontic outcomes. Breastfeeding, bottle-feeding, and mixed-feeding durations were analyzed as separate quantitative exposures, while pacifier duration enabled evaluation of exposure length. The questionnaire underwent multidisciplinary expert review and independent pilot testing, and record eligibility and data cleaning were reported transparently. The statistical analysis used the available denominators, potential confounders, 95% confidence intervals, Firth regression for rare events, and FDR control. This approach distinguished statistically robust findings from nominal exploratory signals.

The principal limitations are the cross-sectional design, convenience recruitment through prematurity-related groups, retrospective parent-reported exposures and outcomes, and absence of exact, clinically verified gestational age. Birth status may have been misclassified, and no independent reliability check against records was possible. The unequal groups (88 preterm versus 20 term) differed in age and potentially in dentition stage; the term-born group is not a representative control population. The feeding-duration analysis included 92/108 children with unambiguous records, and missingness may be selective. The questionnaire underwent expert review and piloting but lacked formal psychometric validation, including for the broad smile item. Small event counts produced some very wide confidence intervals. Residual perinatal, socioeconomic, behavioral, and clinical confounding, plus recall and common-method bias, remain possible. FDR robustness does not confer clinical validity. The findings require prospective replication with verified birth records and standardized clinical assessment.

## 5. Conclusions

No association of feeding duration or reported preterm/term birth status with the evaluated parent-reported orofacial outcomes survived FDR correction. Longer reported pacifier use was associated with a broad, parent-perceived smile abnormality in this sample. This cross-sectional association does not establish causality, identify a specific clinical condition, or determine clinical significance. Prospective studies with verified perinatal data and standardized clinical orofacial assessment are needed.

## Figures and Tables

**Table 1:** Demographic characteristics of children in the primary analysis.

Characteristic	Born Preterm	Born at Term	Total	*p*
Children, n	88	20	108	–
Current age, mean ± SD, years	11.69 ± 3.39	8.80 ± 3.73	11.15 ± 3.62	0.002
Current age, median (IQR), years	12.0 [9.75–14.0]	8.25 [6.0–12.25]	11.5 [8.38–14.0]	0.002
Female children, n/N (%)	49/88 (55.7%)	7/20 (35.0%)	56/108 (51.9%)	0.136

Values are n/N (%) unless otherwise indicated. *p* values were obtained using the Mann–Whitney U test forcurrent age and Fisher’s exact test for sex. IQR, interquartile range; SD, standard deviation.

**Table 2:** Feeding histories and pacifier use reported by parents or caregivers.

Variable	Born Preterm	Born at Term	Total	*p*
Breastfeeding duration, median [IQR], months	6 [1–12] (n = 76)	9 [6–17.25] (n = 16)	6 [2–12] (n = 92)	0.090
Bottle-feeding duration, median [IQR], months	0 [0–12] (n = 76)	0 [0–0.75] (n = 16)	0 [0–12] (n = 92)	0.357
Mixed-feeding duration, median [IQR], months	0 [0–5] (n = 76)	0 [0–1.5] (n = 16)	0 [0–5] (n = 92)	0.586
Any breastfeeding, n/N (%)	59/76 (77.6%)	15/16 (93.8%)	74/92 (80.4%)	–
Any bottle feeding, n/N (%)	28/76 (36.8%)	4/16 (25.0%)	32/92 (34.8%)	–
Any mixed feeding, n/N (%)	26/76 (34.2%)	4/16 (25.0%)	30/92 (32.6%)	–
History of tube feeding, n/N (%)	1/87 (1.1%)	1/20 (5.0%)	2/107 (1.9%)	0.340
Any pacifier use, n/N (%)	60/87 (69.0%)	11/20 (55.0%)	71/107 (66.4%)	0.295
Pacifier duration among users, median [IQR], months	18 [12–24] (n = 54)	15 [12–24] (n = 10)	18 [12–24] (n = 64)	0.505

Feeding and pacifier-use durations are reported in months; 0 months denotes no exposure to that feeding mode. Denominators vary according to available responses. *p* values for durations are from the Mann–Whitney U test; *p* values for binary exposures are from Fisher’s exact test. IQR, interquartile range.

**Table 3:** Parent-reported orofacial outcomes by reported birth status.

Parent-Reported Outcome	Born Preterm, n/N (%)	Born at Term, n/N (%)	Adjusted OR, Preterm vs. Term (95% CI)	*p*	q
Feeding, swallowing, or sucking difficulties	2/82 (2.4%)	1/19 (5.3%)	0.26 (0.03–2.30)	0.227	0.364
Speech articulation difficulties	5/85 (5.9%)	4/20 (20.0%)	0.26 (0.06–1.12)	0.071	0.190
Occlusal or tooth-position concerns	45/85 (52.9%)	14/20 (70.0%)	0.23 (0.07–0.80)	0.021	0.083
Diagnosis of malocclusion	48/84 (57.1%)	13/20 (65.0%)	0.44 (0.14–1.39)	0.162	0.325
Orofacial dysfunction	6/87 (6.9%)	6/19 (31.6%)	0.15 (0.04–0.61)	0.008	0.061
Oral parafunction	13/87 (14.9%)	4/17 (23.5%)	0.55 (0.15–2.06)	0.378	0.504
Perioral/smile abnormality	10/85 (11.8%)	2/20 (10.0%)	1.52 (0.30–7.56)	0.612	0.631
Abnormal oral-health rating	14/86 (16.3%)	3/20 (15.0%)	0.71 (0.18–2.87)	0.631	0.631

Adjusted odds ratios (ORs) compare children reported as born preterm with those reported as born at term; estimates are from Firth logistic regression adjusted for current age and sex. q values were calculated by the Benjamini–Hochberg procedure across eight outcome models; none was below 0.05. CI, confidence interval.

**Table 4:** Associations between reported pacifier-use duration and selected parent-reported outcomes.

Parent-Reported Outcome	n	Adjusted OR per Additional 12 Months of Pacifier Use	95% CI	*p*	q
Perioral/smile abnormality	97	2.29	1.25–4.19	0.007	0.028
Diagnosis of malocclusion	96	1.27	0.82–1.98	0.286	0.381
Occlusal or tooth-position concerns	97	1.29	0.84–1.99	0.240	0.381
Orofacial dysfunction	98	1.03	0.57–1.86	0.913	0.913

Adjusted odds ratios (ORs) are reported per additional 12 months of pacifier use from Firth logistic regression adjusted for current age and sex. Children with an unambiguous report of no pacifier use were assigned 0 months. q values were calculated by the Benjamini–Hochberg procedure across four models. CI, confidence interval.

## Data Availability

Aggregated regional data are derived from publicly available legal acts. Availability of anonymised survey data is subject to ethics approval conditions and institutional policies. The national birth statistics cited in the Introduction are publicly available from Statistics Poland [21].
